# Refining borders of genome-rearrangements including repetitions

**DOI:** 10.1186/s12864-016-3069-4

**Published:** 2016-10-25

**Authors:** JA Arjona-Medina, O Trelles

**Affiliations:** 1 Advanced Computing Technologies Unit, RISC Software GmbH, Hagenberg, 4232 Upper Austria Austria; 2 Department of Computer Architecture, University of Malaga, Campus de Teatinos, Malaga, 29071 Spain

**Keywords:** Synteny block, Computational synteny block, Breakpoint, Refinement

## Abstract

**Background:**

DNA rearrangement events have been widely studied in comparative genomic for many years. The importance of these events resides not only in the study about relatedness among different species, but also to determine the mechanisms behind evolution. Although there are many methods to identify genome-rearrangements (GR), the refinement of their borders has become a huge challenge. Until now no accepted method exists to achieve accurate fine-tuning: i.e. the notion of breakpoint (BP) is still an open issue, and despite repeated regions are vital to understand evolution they are not taken into account in most of the GR detection and refinement methods.

**Methods and results:**

We propose a method to refine the borders of GR including repeated regions. Instead of removing these repetitions to facilitate computation, we take advantage of them using a consensus alignment sequence of the repeated region in between two blocks. Using the concept of identity vectors for Synteny Blocks (SB) and repetitions, a Finite State Machine is designed to detect transition points in the difference between such vectors. The method does not force the BP to be a region or a point but depends on the alignment transitions within the SBs and repetitions.

**Conclusion:**

The accurate definition of the borders of SB and repeated genomic regions and consequently the detection of BP might help to understand the evolutionary model of species. In this manuscript we present a new proposal for such a refinement. Features of the SBs borders and BPs are different and fit with what is expected. SBs with more diversity in annotations and BPs short and richer in DNA replication and stress response, which are strongly linked with rearrangements.

**Electronic supplementary material:**

The online version of this article (doi:10.1186/s12864-016-3069-4) contains supplementary material, which is available to authorized users.

## Background

Large scale genomic rearrangements (LSGR) have been widely studied due to their implication in the evolution of the species. The study of rearrangements is strongly linked with Synteny Blocks (SB) defined as conserved regions between sequences [1]. The regions between SB are called breakpoints (BP), and their study might reveal clues towards evolutionary mechanisms [2, 3]. Both, SB and BP, have been used for phylogeny distance calculation [4], ancestral genome reconstruction [5], and others.

Although there are many methods to identify SBs, they usually do not refine their borders [3, 6, 7]. Those methods that refine SBs -and therefore BP- they usually focus on extending the borders of the SB, aiming to maximize a specific target function based on the alignment. Additionally, the lack of a well-accepted definition of SB [8] might be among the reasons that current tools yield widely different results. Furthermore, the presence of repeated regions or small blocks between the SBs increases the complexity of the detection, one of the main reasons why most methods do not take into account such repetitions. However, these repetitions -mostly associated with mobile elements- have been driving the evolution in many ways [9].

One of the main problems to identify BPs is the unclear definition. For example, Lemaitre et al. [10] reasoned that a BP is not a single “point” but a region between two SB; while others, for example Chu et al. [11] describe a method to determine the exact location of a BP at nucleotide level for inversions and block interchange events.

A second problem appears when trying to refine the SB by extending its borders. Current methods try to maximize the alignment in the region between two SBs, but boundaries are less conserved. Most of them [12–14], remove the small blocks or repetitions to simplify the SB detection. Clearly the resulting BPs might be contaminated by small subsequences which actually have a homologous region in the other sequence. Any analysis based on these contaminated sequences will be biased by these small subsequences.

In a recent work [15] we addressed the detection of blocks of large rearrangements, called Computational Synteny Blocks, taking into account repetitions. In this manuscript, we propose a method to refine these detected CSBs and detect also BPs taking into account small blocks and any kind of repetitions. Indeed, we use the repetitions alignment to improve the accuracy of the refinement process. In our model, we contemplate inversions, duplications and translocations.

Our results show a higher accuracy in terms of percentage of identity in refined SBs. Our results also indicate biological differences between refined SBs and detected BPs sequences. Sequences in the SBs borders are richer in DNA damage whereas sequences in the detected BPs are richer in DNA replication and stress response, strongly linked to evolution [16].

## Methods

**Fig. 1:**
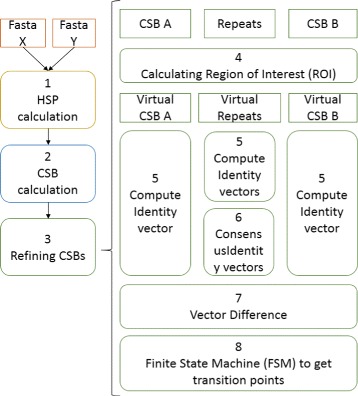
Workflow from fasta sequences to refined blocks and BP detection

Our method starts with the collection of Computational Synteny Blocks (CSB) - similar to SB associated with coding regions, and CSB also covering non-coding regions. The CSBs are calculated using GECKO-CSB [15] (second step in Fig. 1). Applying linearity and collinearity functions (described in [15]) over the CSB provided by GECKO-CSB we identify LSGR (so far duplications, inversions and translocations). The next step — which is reported in this document- is the precise refinement of the borders of CSBs involved in every detected LSGR (third step in Fig. 1). This refinement is applied to the sequences involved in calculation (namely sequences *X* and *Y*) in two independent and separable processes. After that we combine the results to get the final refinement. Figure 1 describes the workflow step by step.

**Fig. 2:**
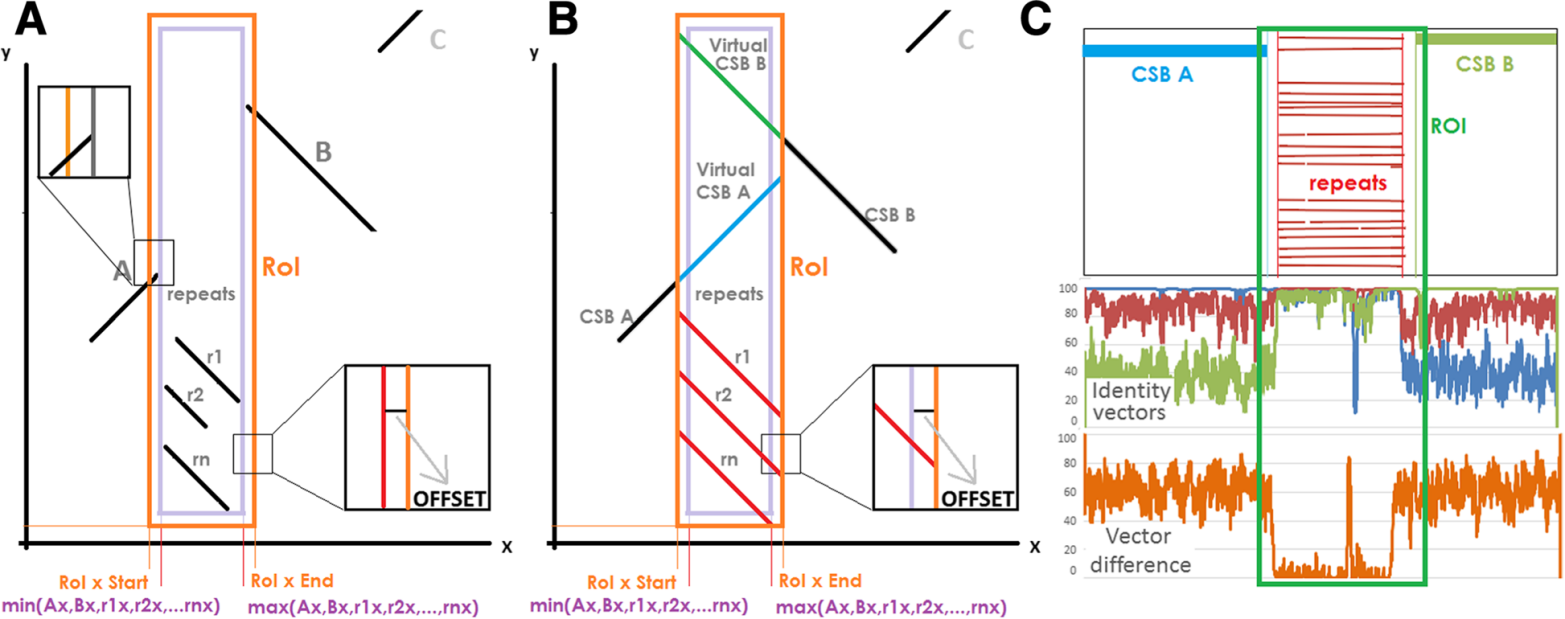
Illustrative representation of the Region of Interest (ROI). **a** ROI region in an inversion event (CSB B). Coordinates are calculated following the Eqs. 1 (**b**) Virtual CSBs and repetitions. Virtual CSBs are calculated using the Eqs. 2 (**c**) Same representation but including identity vectors and vector difference graphs

Once an LSGR is detected, we take the two CSBs involved. The repetitions in between them, if any, are also take into account. Then we define a region of interest (ROI) running from the tail of one CSB to the head of the other (step 4 in Fig. 1). This ROI includes an arbitrary *offset* to force the overlapping between CSBs and repetitions (see Figs. 2 and 12). A virtual CSB (*C* *S* *B*_*V*_) and virtual repetitions are created by extending the borders in order to cover the ROI. Afterwards, these *C* *S* *B*_*V*_ and virtual repetitions are aligned using a fast customized implementation of the Needleman and Wunsch [17] global alignment method. The main idea of this process is to force overlapped regions to study the alignments within the ROI.

At this point an identity vector for every aligned *C* *S* *B*_*V*_ and all repetitions is computed (step 5 in Fig. 1. See Additional file 1 for more details). Then, a “difference vector” (*V*_*diff*_) is calculated (step 7). If we are working with only two *C* *S* *B*_*V*_, the *V*_*diff*_ contains the normalized absolute difference between the two identity vectors. If besides that we are working with repetitions, we compute the *V*_*diff*_ taking into account a consensus identity vector from the repetitions (step 6).

The rationale behind the method is the following: The *V*_*diff*_ vector contains high values when identity vectors are different. In those regions where values are similar in both identity vectors, the values contained in *V*_*diff*_ will be low. At some point we will observe a transition between high and low values along the *V*_*diff*_ vector. These transitions will define the BP. A finite-state machine (FSM) was designed to detect these transitions (step 8). At the end of the process, CSB borders are refined based on the BPs detected by the FSM. The method does not force the BP to be a region or a point. This will depend on the transition’s features.

### Detection of CSBs repetitions and large-scale genomic rearrangements

CSBs and repetitions are detected using Gecko-CSB [15], an extension of Gecko [18]. This software has demonstrated its capacity to yield HSPs of high-quality beating reference software. In [15] we presented a set of formal definitions describing different levels of linearity and collinearity between CSBs. Using these definitions, a set of rules was defined to identify LSGR in single chromosome species, such a inversions, translocations, reverted translocations and duplications. Once a LSGR is detected, we perform our refining method over those CSBs involved in the LSGR.

After the detection of a LSGR two CSBs (namely *A* and *B*) are selected. Optionally, if collinearity between CSB *A* and CSB *B* is interrupted by a set of repeats, the repeats will be included in the selection as well. Repeats can be separated in two groups. Those repeats whose coordinates in the sequence *X* overlap with CSBs *A* and *B* are grouped in a collection named repeats-*X*. In the collection repeats-*Y* are the equivalents regarding sequence *Y*.

### Refining CSBs

At this point the method splits in two branches. The refinement in the sequence *X* and *Y* are complementary and independent. In this document we will describe the refinement for the sequence *X* branch. The sequence *Y* branch is the same, but interchanging *X* by *Y*.

### Calculating the region of interest

The CSBs and repeats define a ROI (see Eq. 5, Figs. 2 and 12). Since our method is focused on finding transitions between CSBs and repetitions, we introduce an *offset* parameter, which ensures overlapping between the end of CSBs and the beginning of virtual CSBs and the virtual repetitions, guaranteeing that transitions are present. In the worst case, the method will have *offset* number of nucleotides in both CSBs that share similarity and therefore, they can be aligned with a high value of identity. In other words, the *offset* parameter stabilises the beginning and the end of the signal (More details in “FSM thresholds selection” in the Additional file 1). The ROI is defined as follows:

1 $$  {}\begin{aligned} ROI_{xStart} &= min(A_{xEnd}, B_{xStart}, Repeats_{xStart}) - {offset} \\ ROI_{xEnd} &= max(A_{xEnd}, B_{xStart}, Repeats_{xEnd}) + {offset} \\ ROI_{yStart} &= min(A_{yEnd}, B_{yStart}, Repeats._{yStart}) - {offset} \\ ROI_{yEnd} &= max(A_{yEnd},B_{yEnd},Repeats_{yEnd}) + {offset} \end{aligned}  $$

After calculating the ROI, new CSBs named virtual CSBs (*C* *S* *B*_*V*_) are created using the ROI *X*_*Start*_ and *X*_*End*_ coordinates. This means that all *C* *S* *B*_*V*_s will start and end at the same point. In this step we are extending or trimming the old CSBs concerning ROI start and end points. New *C* *S* *B*_*V*_s’ *Y* coordinates will be calculated depending on how much we have trimmed or extended the coordinates in *X* regarding the old CSB. The equations that describe this process are the following:

2 $$  \begin{aligned} {CSB}_{V xStart} &= ROI_{xStart} \\ {CSB}_{VxEnd} &= ROI_{xEnd} \\ \alpha_{L} &= CSB_{xStart} - {CSB}_{VxStart} \\ \alpha_{R} &= {CSB}_{VxEnd}- {CSB}_{VxEnd} \\ {CSB}_{VyStart} &= CSB_{yStart} - \alpha_{L} \\ {CSB}_{VyEnd} &= CSB_{yEnd} + \alpha_{R} \end{aligned}  $$

Notice that *α* takes negative values when trimming and positive when extending. New *C* *S* *B*_*V*_s are aligned using a Needledman and Wunsch implementation.

### Calculating identity vectors

After the alignment of *C* *S* *B*_*V*_s, identity vectors (*I*_*V*_) are created for every *C* *S* *B*_*V*_. All *I*_*V*_s have the same length and they represent the percentage of identity that a certain region of length *W* has in the alignment. We take a window of length *W* to calculate that percentage of identity.

First we create a binary vector (*V*_*B*_) which represents matches in the alignment. *V*_*B*_ has the length of the alignment. Since *V*_*B*_ takes into account gaps, its length can be different from one *C* *S* *B*_*V*_ to another. By using a window of length *W*, we can compute the percentage of identity at any point in *V*_*B*_. As long as we are going to compare *I*_*V*_ from different *C* *S* *B*_*V*_s, identity values from those points in the alignment that represent a gap in sequence X are not stored. This way, all identity vectors from different *C* *S* *B*_*V*_s will have the same length, *R* *O* *I*_*length*_.

Low values in parameter *W* produce a noisy identity vector corresponding with high frequency changes of identity. On the contrary, high values in parameter *W* smooth the noise and produce a low frequency signal. The selection of a proper *W* value is not possible as it might change depending on the *C* *S* *B*_*V*_ involved. We could also be interested on changes that happen at different frequencies. Therefore, instead of choosing a fixed *W* value, which would mean changes at only one frequency, we build a vector containing all frequencies as follows:

3 $$  I_{V}(x) = \sum\limits_{i=0}^{N} A_{i}I_{i}(x)  $$

where *A*_*i*_ is the weight of the identity vector at a certain frequency

4 $$  \sum\limits_{i=0}^{N} A_{i} = 1  $$

And the Identity vector at a certain frequency is calculated as follows:

5 $$  I_{i}(x) = \frac{1}{2N+1}\sum\limits_{j=x-N}^{x+N} V_{B}(j)  $$

In this model, *N* defines the maximum window to compute the percentage of identity and also defines the start and end positions where the values of the vector can be used. From 0 to 2*N*+1 and from 2*N*+1−*R* *O* *I*_*length*_ to *R* *O* *I*_*length*_ the *I*_*V*_ is uncompleted. Therefore, *N* cannot be as long as we want. It should be at least lesser than *OFFSET*. In practice we have observed that a value of 50 is enough to get good results.

Finally, since identity vectors are going to be compared, they must to be normalized.

### Calculating consensus identity vector

In the case that a group of repetitions are detected, we use the information of the consensus sequence to improve accuracy of the refinement method.

After repeats have been aligned and the *V*_*B*_s have been computed, a Sum Match Vector (*V*_*SM*_) is calculated by adding all *V*_*B*_s vectors. This vector has a length of *R* *O* *I*_*length*_, so only positions which are not representing a gap are taken into account -as we did in the previous section. Then, we calculate the percentage of repeats that cover one specific position in the *V*_*SM*_. To calculate the Consensus Identity Vector (*V*_*CI*_), only positions that comply with a given threshold are set to 1, and 0 otherwise. In this implementation the threshold was set to 25 %. This new vector is named Consensus Binary Vector. After this process, we calculate the *V*_*CI*_ by processing the Consensus Binary Vector as we already described in the previous section.

### Vector difference

In order to detect transitions which delimitate the BP, we compute the absolute difference between the *C* *S* *B*_*V*_s identity vector. *C* *S* *B*_*V*_s are extracted from CSBs according to the *ROI*, using the *OFFSET* to ensure that similar regions are represented in *C* *S* *B*_*V*_s. As a result, the identity vectors for the *C* *S* *B*_*V*_-A have a high value at the beginning and low value at the end. On the contrary, the identity vectors for the *C* *S* *B*_*V*_-B have a low value at the beginning and high value at the end. This is the reason why the vector difference will start and end with high values. If repetitions are detected, then the difference vector will have high values in the middle as well.

Anyways, transitions will be found in between these high values (see Fig. 2).

### Detecting transition points

**Fig. 3:**
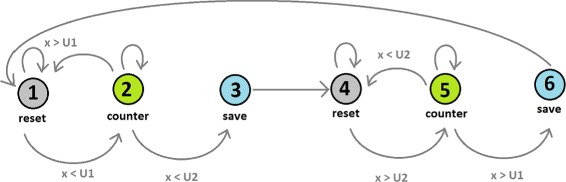
Finite State Machine to detect transitions. This FSM has six states. The first three states (*1–3*) are to calculate the BP’s start coordinate, and the last three states (*4–6*) to calculate the BP’s end coordinate. Changes from one state to another will depend on vector difference values (x in the figure) and thresholds U1 and U2

To detect transitions a Finite-State Machine (*FSM*) was designed. Figure 3 shows the design. Basically, the *FSM* detects the coordinates where the vector difference value was the last time at a certain threshold (*U*1) before reaching the second threshold (*U*2). As a result, the selected region defined by the coordinates is the transition between high and low values along the vector difference.

We associate these transitions as a candidate for a BP. After this process, the refined *SB* can be trimmed or extended. The threshold selection is discussed in the next section.

## Results

### Simple case

We will use a simple case to illustrate the algorithm behaviour in the SB borders-refinement method using M. *hyorhinis* HUB-1 (Accession code NC-014448.1) and M. *hyorhinis* SK76 (Accession code NC-019552.1) genome sequences with a length of 839,615 bp and 836,897 bp, respectively.

**Fig. 4:**
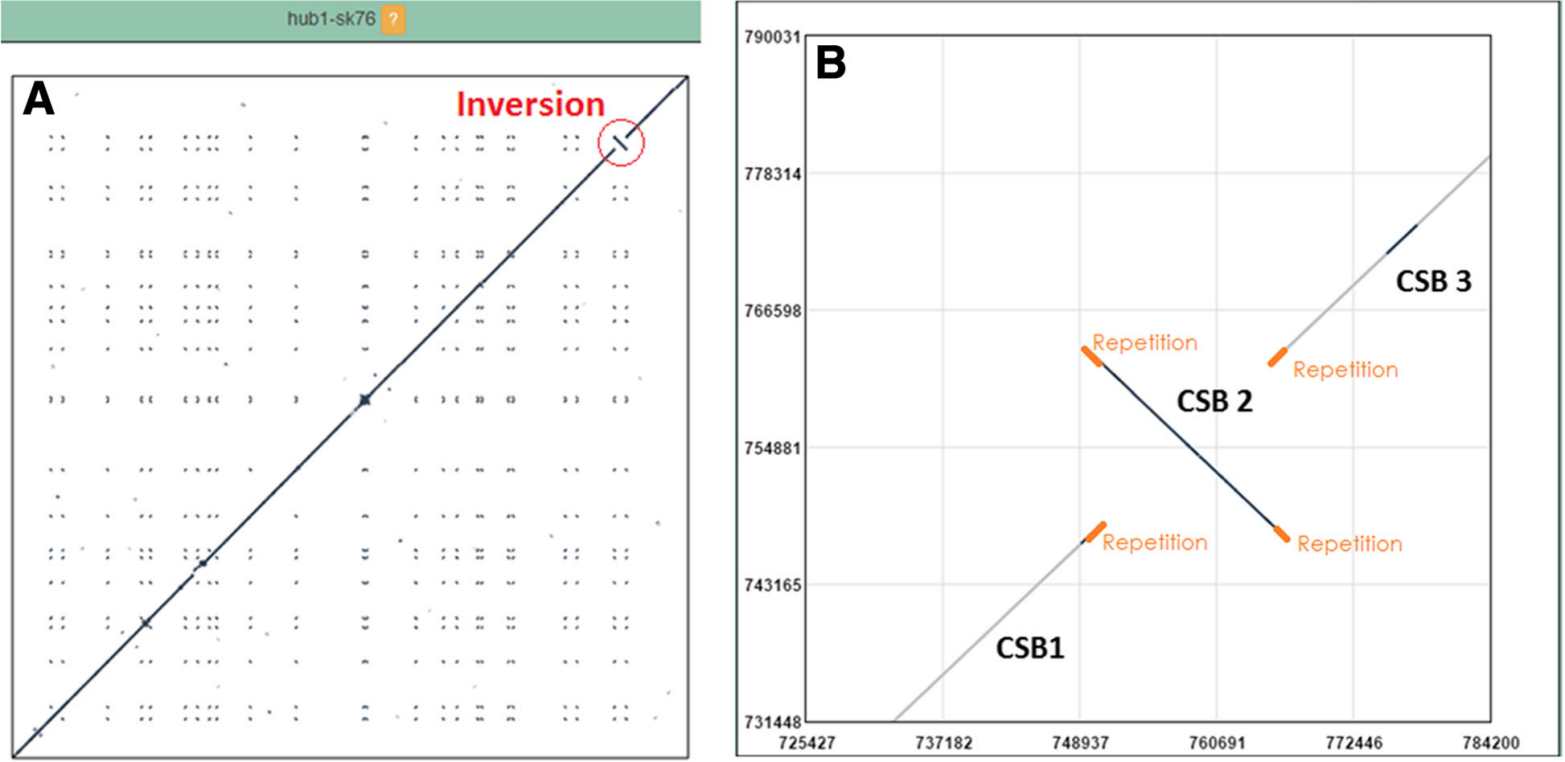
**a** Full comparison of HUB-1 against SK76. Main diagonal represents that both subspecies are quite similar. Small points represent repetitions, with a notorious one (an inversion) present upper zone of the image (*circle*) (**b**) Zoomed display of the marked region in 4a. Three CSBs are going to be extended in this example. Repetitions are represented in a different colour

Figure 4 a shows the full comparison of HUB-1 against SK76. Figure 4 b shows a particular area where a LSGR (an inversion) is presented, before the refinement.

Table 1 shows the coordinates of the CSBs involved in the inversion before and after the refinement process, where *X* represents M. *hyorhinis* HUB-1 and *Y* correspond to M. *hyorhinis* SK76. Str. column represents the strand of the *Y* sequence, forward or reverse. The percentage of extension in *X* and *Y* sequence is shown in *Δ* *X* and *Δ* *Y* columns.

**Table 1:** CSB Coordinates before and after the refinement process

	*X*_*Start*_	*Y*_*Start*_	*X*_*End*_	*Y*_*End*_	Str	Length	% *Δ* *X*	% *Δ* *Y*	% ident	
*C* *S* *B*_1_										
Before	711,591	710,528	747,965	746,902	f	36,379			99.69 %	
After	711,591	710,528	748,001	746,940	f	36,413	0.1 %	0.1 %	99.72 %	
*C* *S* *B*_2_										
Before	749,573	761,860	762,895	748,534	r	13,348			99.37 %	
After	749,564	761,853	762,933	748,505	r	13,349	0.35 %	0.17 %	99.59 %	
*C* *S* *B*_3_										
Before	764,581	763,521	780,474	779,414	f	15,895			99.70 %	
After	764,494	763,439	780.474	779.414	f	15,976	0.55 %	0.52 %	99.69 %	

The percentage of identities has increased a bit due to the extension (the refined CSBs are a bit longer). Notice that in *C* *S* *B*_2_ the refine process has extend the *Y*_*Start*_ coordinate making the CSBs 7 nucleotides shorter. On the other hand, in the opposite border (*y*_*End*_) it has extended 29 nucleotides.

Four regions have been detected as repeated sequences. A database search (Uniprot bacteria at ftp://ftp.ebi.ac.uk) using SMA3s [19] was carried out. Results and sequence features are shown in Table 2.

**Table 2:** Repeated region coordinates

ID	Sequence	Start	End	Length	Description	Enzyme
1.x	*hyorhinis HUB-1*	748,012	749,513	1,501	tnp	Transposase
2.x	*hyorhinis HUB-1*	762,953	764,494	1,541	insK	Integrase core domain
						protein
1.y	*hyorhinis SK76*	746,988	748,493	1,505	tra	Transposase for insertion
						sequence element IS6290
2.y	*hyorhinis SK76*	761,936	763,425	1,489	insK	Mobile element protein

And the BPs are shown in the Table 3.

**Table 3:** Breakpoint coordinates

ID	Sequence	Ref seq	Start	End	Length
1.1a	M. *hyorhinis* HUB-1	NC-014448.1	748,001	748,012	11
1.2a	M. *hyorhinis* HUB-1	NC-014448.1	749,513	749,564	51
2.1a	M. *hyorhinis* SK76	NC-019552.1	746,940	746,988	48
2.2a	M. *hyorhinis* SK76	NC-019552.1	748,493	748,505	12
3.1a	M. *hyorhinis* HUB-1	NC-014448.1	762,933	762,953	20
3.2a	M. *hyorhinis* HUB-1	NC-014448.1	764,494	764,539	45
4.1a	M. *hyorhinis* SK76	NC-019552.1	761,853	761,936	83
4.2a	M. *hyorhinis* SK76	NC-019552.1	763,425	763,439	14

In this case the method has found 8 BPs. Due to repetitions that the method detects between two CSBs, two BPs are detected in each sequence. For each BP found, we have performed a database search using Uniprot and NCBI non-redundant with no results. No annotation was found.

#### Comparing with CASSIS software

We have processed the CSBs detected by GECKO-CSB using CASSIS [12] in order to refine them. Since CASSIS cannot handle repetitions and following the recommendations from its article, we have masked all the repetitions in both sequences using RepeatMasker [20] (search Engine was *abblast*) and we did not include the repetitions in the input file. Data set and results can be found in the Additional file 1.

Results from CASSIS are widely different than those obtained by our method because, among other reasons, they do not take into account repetitions. Our method detects 2 short BPs where CASSIS detects a big one. Indeed, BP 3b and 4b (SK76 sequence) cover the region contained by CSBs 1, 2 and 3. This result is incomprehensible because it implies that the SBs desapear, creating a huge BP of size around 85 Kbps, instead of these 3 SBs.

BP 1b has a length of 1,608 bps. We have performed a BLAST [21] search using the sequence of BP 1b with default parameters. The sequence has been found several times in different sub species of *hyorhinis* with high values of identity and coverage, which point-out that this sequence is a part of a conserved repetition (see BLAST Report-BreakPoint-1b in Additional file 1). An additionally BLAST search was carried out using sequences from BP 2a with similar results.

We have performed a database search using SMA3s over the BP detected by CASSIS. Results are shown in Table 4 (description and enzyme columns).

**Table 4:** CASSIS software breakpoint coordinates

ID	Sequence	Start	End	length	Descript.	Enzyme
1b	M. *hyorhinis* HUB-1	747,965	749,573	1,608	tnp	Integrase core domain protein
2b		762,895	764,581	1,686	insK	Integrase core domain protein
3b	M. *hyorhinis* SK76	710,797	797,477	86,680	polC	DNA polymerase III PolC-type
4b		712,895	797,477	84,582	nanE	ManNAc-6-P epimerase

### Testing the method with a 68 mycoplasmas dataset

For the next test, a collection of 68 Mycoplasmas was used. This test was performed with the aim to avoid bias in the analysis that a selection of two particular genomes could introduce. The genome collection and their gene bank annotations are available at http://bitlab-es.com/gecko/. For the biological analysis we have performed SMA3s [19] over the sequences to find annotations using the Uniprot bacteria database (ftp://ftp.ebi.ac.uk). Additionally blast2GO [22] was used to carry out a second annotation process using blastx and the non-redundant protein database filtered by Bacteria taxa.

We run first GECKO [18] over the resulting 2,278 comparisons following by GECKO-CSB [15]. After that, the refinement process was carried out giving the refined collection of CSBs as a result.

**Fig. 5:**
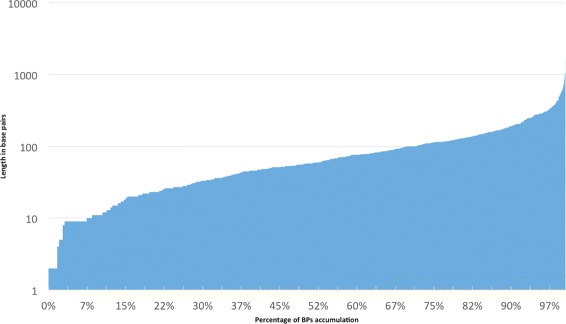
Progressive distribution of Breakpoint length (bps)

**Fig. 6:**
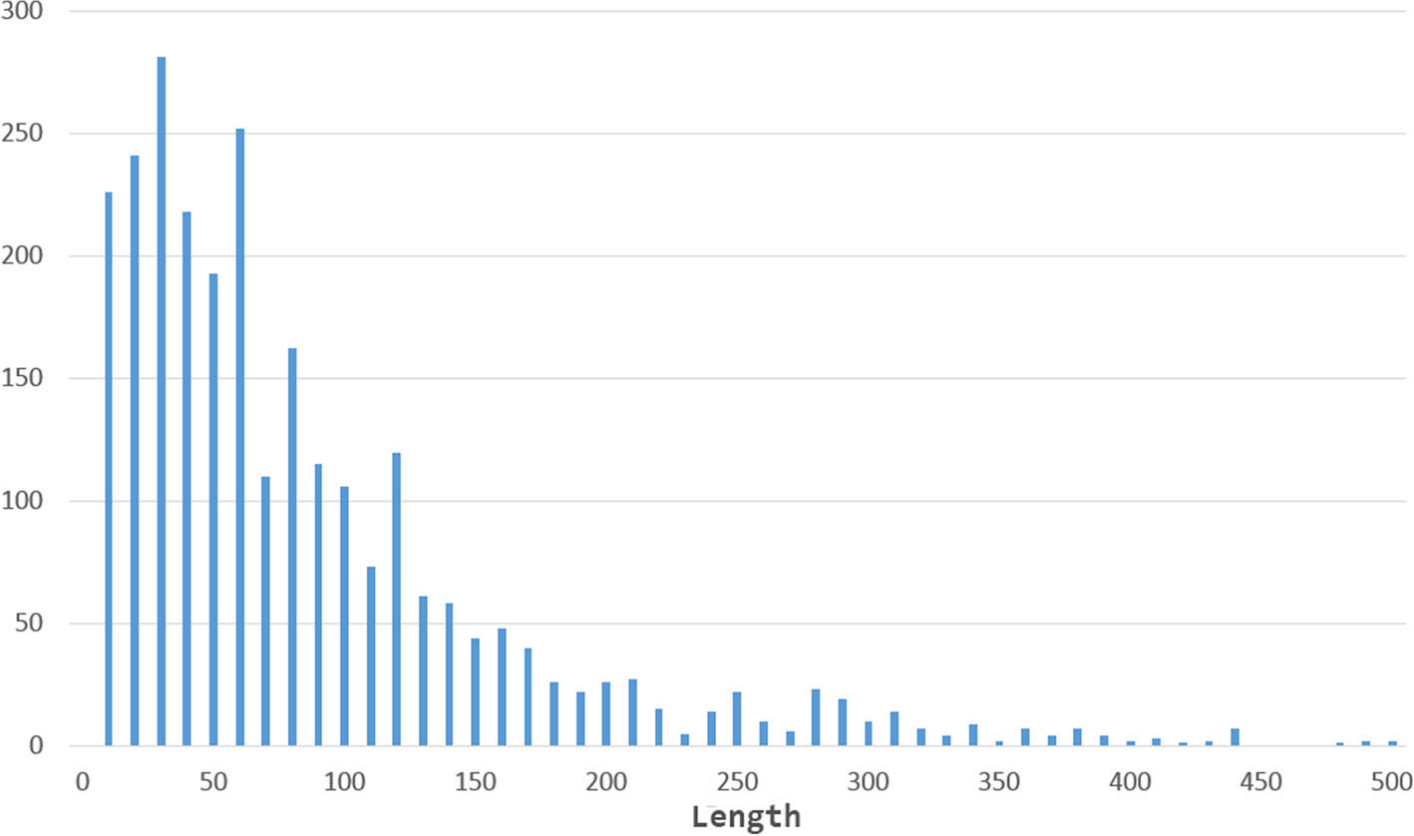
Frequency distribution of Breakpoint length

Our method refined 2,213 CSBs, 829 were trimmed after the refining process and 1,384 were extended. Around 70 % of the BPs detected are sized below 100 bps, 95 % below 300 bps (see Fig. 5). The BP detection was limited in the implementation at a size of 5000 bps to avoid spurious long BPs. As it can be observed in Fig. 6, the frequency of the length tends to zero at length of around 400 bps.

To analyse the results from a biological point of view, BPs sequences were extracted. The sequences of the proportional region of the adjacent Synteny Block (PRASB) of each BP were also extracted according with the BP length (the length of the PRASB sequence has the same length of the BP sequence, see Fig. 12). The purpose was to find biological differences by comparing results from annotations in BP and PRASB sequences. The sequences were compared against the NCBI non-redundant protein database, filtered by Bacteria taxa. After that, the sequences were mapped and annotated using blast2GO [22].

**Fig. 7:**
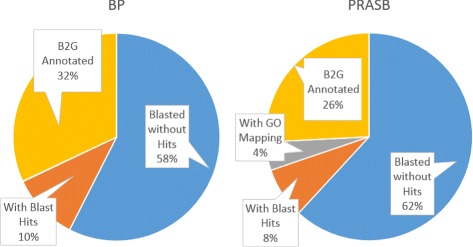
Results of blast search, mapping and annotation process with blast2GO for BP and PRASB sequences

**Fig. 8:**
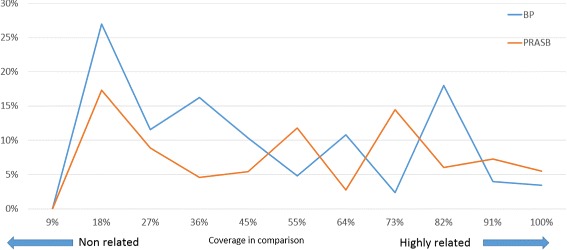
Percentage of annotated sequences in BP and PRASB by percentage of coverage in the comparisons in which the sequences were extracted from

The number of sequences with annotation was higher in BPs (32 %) than in PRASBs (26 %). For more details, see Fig. 7. We also analysed the percentage of annotations by level of coverage that cover the CSBs in the comparison from which the BPs were detected. We found that at a lower level of coverage (meaning non related species), more sequences were annotated, especially in BPs sequences (27 % vs 17 %, see Fig. 8).

**Fig. 9:**
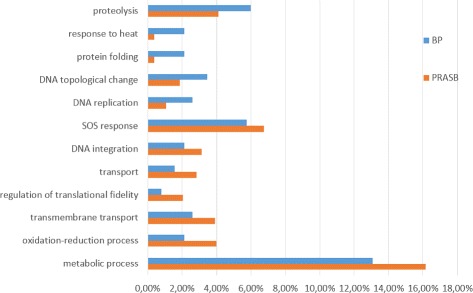
Results from Blast2GO for biological process in annotations of BP and PRASB sequences. Percentages are over the total amount of annotations

Regarding the content of the annotation, we found several differences in the biological process and molecular function categories. Figure 9 shows a summary of the biological process category with the most significant differences between BPs sequences and PRASBs sequences. SOS response, DNA integration or metabolic process were more present in PRASB sequences. Proteolysis, response to heat, protein folding, DNA topological change and DNA replication were found in more proportion in BP sequences. Full reports are available as Additional file.

**Fig. 10:**
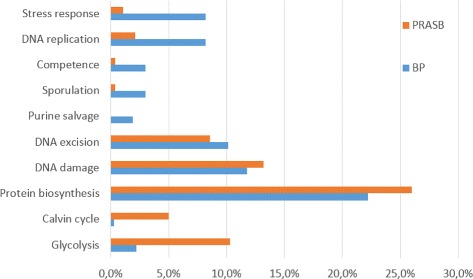
Results of Uniprot keyword categories for biological process in annotations of BP and PRASB sequences. Percentages are over the total amount of annotations

We also performed another database search, which was carried out using SMA3s [19] against the UNIPROT database. The results showed strong differences between annotations in BPs and PRASBs sequences. Figure 10 shows the UNIPROT keyword categories for Biological process. Stress response and DNA replication are more present in BP sequences. On the other hand, Glycolysis, Calvin cycle and DNA damage are significantly more present in the PRASB than in BP sequences.

**Fig. 11:**
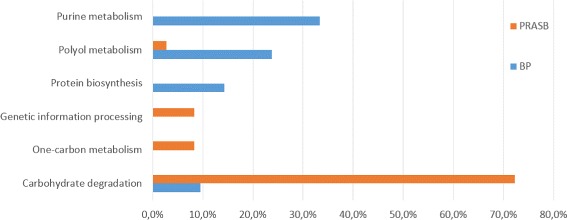
Results of Uniprot pathways in annotations of BP and PRASB sequences. Percentages are over the total amount of annotations

Figure 11 shows the UNIPROT pathways. Carbohydrate degradation is by far more represented in PRASB sequences and Purine metabolism is more present in BP sequences. Full reports are available as Additional file.

**Fig. 12:**
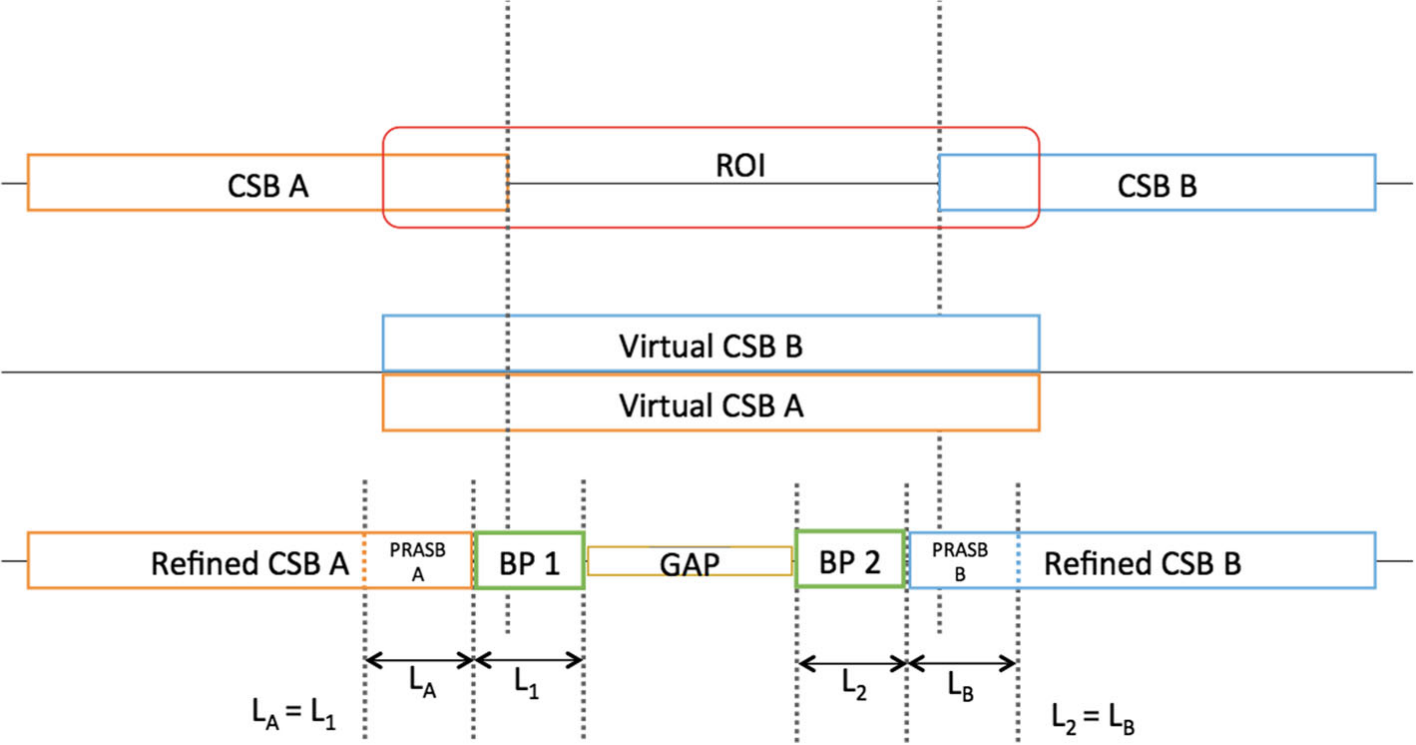
CSBs before and after the refinement. At the end of the refinement process, we detect BPs. We also extract PRASB and GAP sequences to analyse accuracy of the method. PRASB and BP have the same length

The method we present in this manuscript detects two BPs when refining SBs, one at each border (tail or head) of the SB, instead of considering the whole region between these SBs as one large BP. Therefore, after the refining process we have two BPs and one region in between (gap), as it can be observed in the Fig. 12. The sequences corresponding with this region in between the BPs were extracted to be analysed.

Around 30 % of the gap regions in between two breakpoints are shorter than 100 bps of length, 88 % below 1,000 bps.

In order to analyse biological differences between BPs and the gap between two BPs once SBs borders have been refined, we have extracted the sequences corresponding with the gap regions between BPs.

**Fig. 13:**
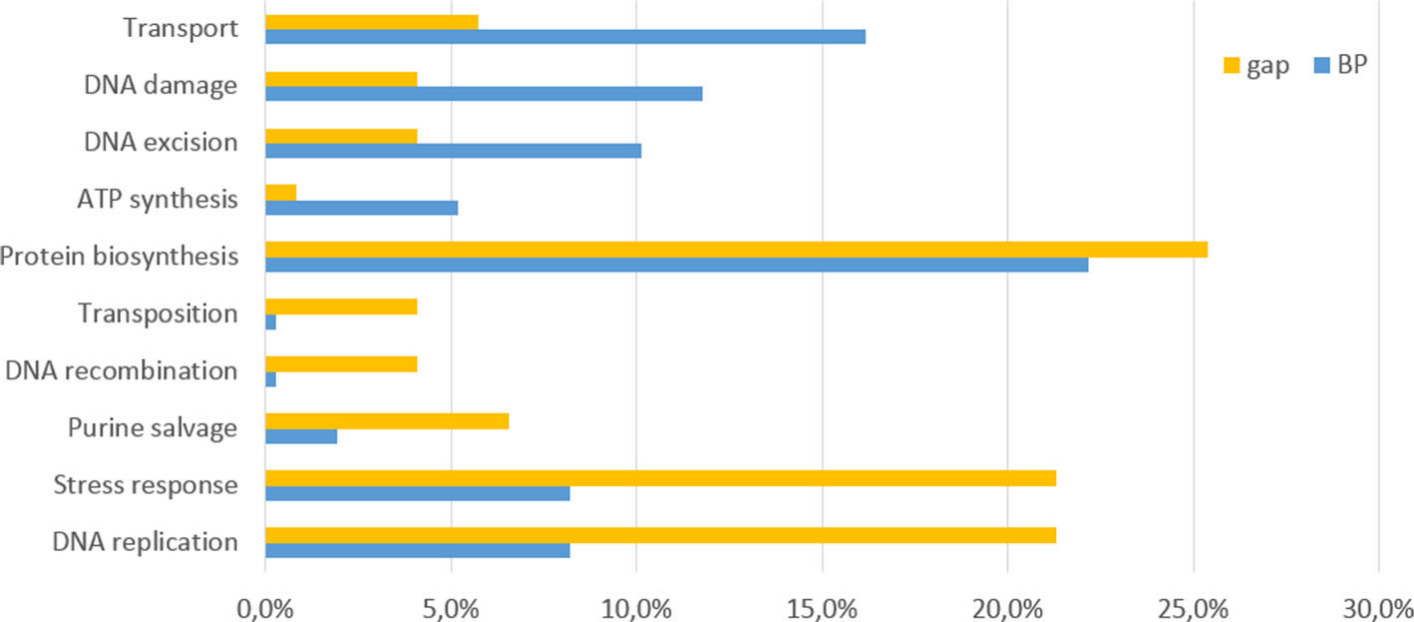
Results of UniProt keyword categories for biological process in annotations of BP and gap sequences. Percentages are over the total amount of annotations

A SMA3s search was carried out over BPs sequences and the gap sequences using the Uniprot database. The main difference according with these results is at the biological process (Fig. 13). DNA replication, Stress response and Purine salvage were found more often in the gap whereas transport, DNA damage and DNA excision were more present in the BP sequences.

## Discussion

### The break point definition

A SB is defined as a relation between two conserved regions in the sequence of two different species, in terms of homology or similarity. A BP is usually known as the region in between two SBs that have suffered a rearrangement due to a LSGR. Many studies support that LSGR do not happen randomly but follow an unknown model. Some regions of the sequence seem to be more fragile or predispose to suffer a large-scale LSGR [2]. Indeed these BPs can be reused [3, 23] and the BP reuse rate is strongly linked with the resolution in which SB are detected [24].

Therefore, if a BP depends on the “fragility” of the specific regions in the sequence then it should not be defined as a relation between two specific regions of two sequences (as SB is defined). Although so far a comparison method is needed to detect them.

Current methods based on sequence comparison, detect SBs by joining or chaining High Score Segment Pairs, and when they refine their borders, they try to expand the SB borders by maximizing a target score function. This means that the BP region will be a region without similarity. However, following the previous reasoning about BP definition, it implies that BPs regions do not have to be necessarily regions with almost no similarity. Two species could share the same BP and therefore, the sequences would have some level of similarity. We think that when refining SBs, they can be trimmed as well as expanded after the refinement process.

### Threshold selection in the finite state machine

**Fig. 14:**
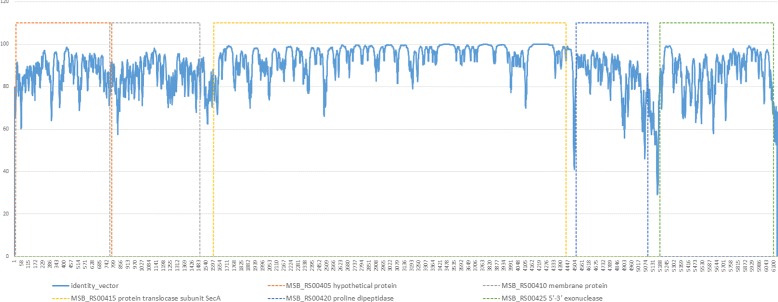
Real case of SB identity vector. In dotted lines codding regions for sequence X. SB extracted from NC-014751.1 (sequence X) vs NC-015431.1 (Sequence Y) comparison. *X*_*Start*_: 92,877, *Y*_*Start*_: 115,660, *X*_*End*_: 98,983, *Y*_*End*_: 121,755

Our method bases the BP detection on transition points in the differences of the percentage of identities. We have analysed the behaviour of the identity vector along SBs. We have found that coding regions and non-coding regions have different levels of identity, which can be explained because of different evolutionary level of pressure. But we also have found that in many cases there is a perceptible transition that could be detectable using a FSM (see Fig. 14). We think that something similar might happen between SBs and BPs, a detectable transition that could determine the BP region.

To identify these transitions we have designed a FSM which uses two thresholds. In the current version of the implementation of the method, which we have described in this document, thresholds are set to 80 and 20 respectively. The selection of the parameter values was made empirically. (see “FSM thresholds selection” in the Additional file 1 for more details).

**Fig. 15:**
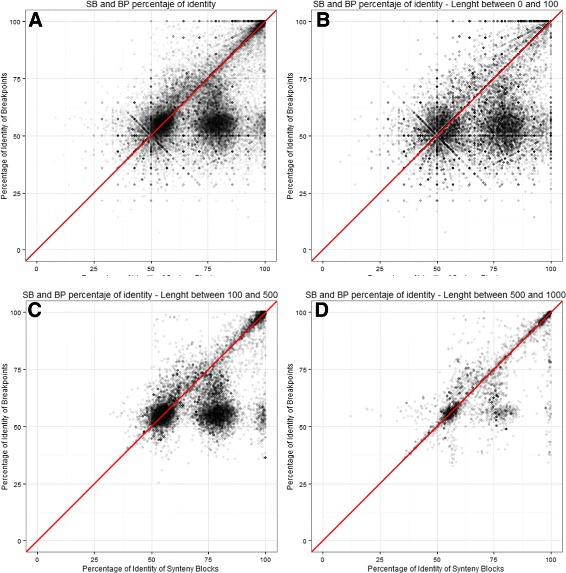
Percentages of identities in SB (axis x) and BP (axis y) regions. **a** all pairs of SBs and BPs. **b** only BPs with length between 0 and 100. **c** length between 100 and 500. **d** length between 500 and 1,000

We analysed the identity percentage of SBs and BPs at different length and have found a strong correlation between SB and BP levels of identity percentage (see Fig. 15). In general BPs have less identity percentage than SB.

## Conclusions

We have developed a method to refine the borders of CSBs taking into account repetitions and using them to improve the accuracy of the refinement. The method is not based on maximizing any target function, but studies the alignments to refine and uses a finite-state machine to find transition points in the alignment. These transition points set an accurate refinement of the involved blocks. Due to the method’s features, BPs are detected as regions or as points, depending on the specific case. It also takes into account the repeated regions, so between two CSBs it can give 4 breakpoints, 2 for each sequence, demarcating start/end of one block and end/start of the region in between.

Several analysis were carried out in order to find biological differences between BPs, SBs borders and gap regions.

The results showed that there are biological differences between BPs sequences and the PRASB sequences. BPs sequences are biologically richer than PRASB. Both searches using Uniprot and NCBI databases gave more results in BPs sequences than the PRASB sequences. However, PRASB showed more diversity in annotations than those obtained for BPs.

Our experiments show that there may to be a correlation between the number of sequences annotated in BPs and PRASB and the relatedness of the species from which those sequences were extracted.

We have also found that there are differences between what we consider as BPs and the region in between the BPs, whereas other methods just consider the whole region as BP.

Our method needs two thresholds to detect the transition points in the difference vector in which the BP is defined. Thresholds pick up the abrupt changes in the signal. These thresholds are fixed in this version of the method, however, we will work on a dynamic configuration of the threshold based on SB similarity that might produce more accurate results.

## Additional file

Additional file 1 Supplementary material. (PDF 2796 kb)
