# Supplementary material for: Refining borders of genome-rearrangements including repetitions
Source: BMC Genomics. 2016 Oct 25;17(Suppl 8):804. doi: 10.1186/s12864-016-3069-4 (PMC5088515; doi:10.1186/s12864-016-3069-4)
Supplement: Supplementary file 1 — Supplementary material. (PDF 2796 kb) [file 12864_2016_3069_MOESM1_ESM.pdf]

Refining borders of genome-rearrangements using repetitions
Supplementary Material

J. A. Arjona-Medina, O. Trelles

Calculating identity vectors

After the alignment of roiCSBs, identity vectors are created for every roiCSB. All Identity vectors have the same length and they represent the percentage of identity that a certain region of length W have in the alignment.

First we create a binary vector (Vm) which represents matches in the alignment.

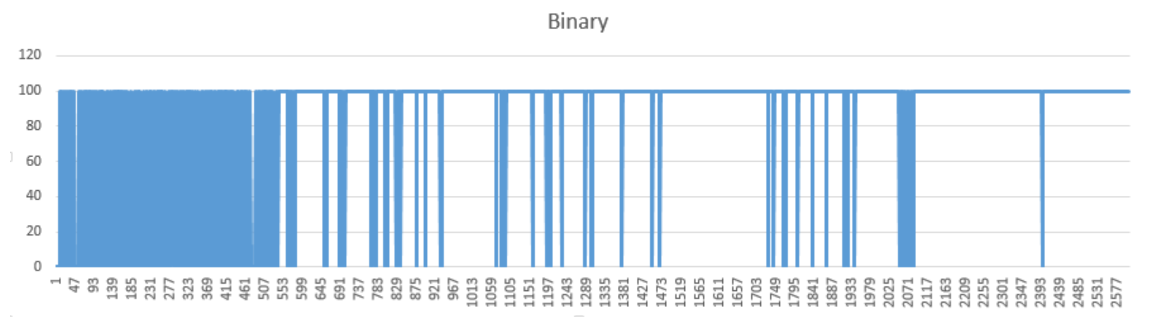

Figure 1. Binary vector from alignment

Vm has the length of the alignment. Since Vm takes into account GAPs, its length can be different from one roiCSB to another. By using a window of length W, we can compute the percentage of identity at any point in Vm. As long as we are going to compare identity vectors from different roiCSB, identity values from those points in the alignment that represent a GAP in sequence X are not stored. This way, all identity vectors from different roiCSB will have the same length, ROI_length.

Low values in parameter W produce a noisy identity vector corresponding with high frequency changes of identity. On the contrary, high values in parameter W smooth the noise and produce a low frequency signal.

Figure 2 shows some identity vectors using different values of W.

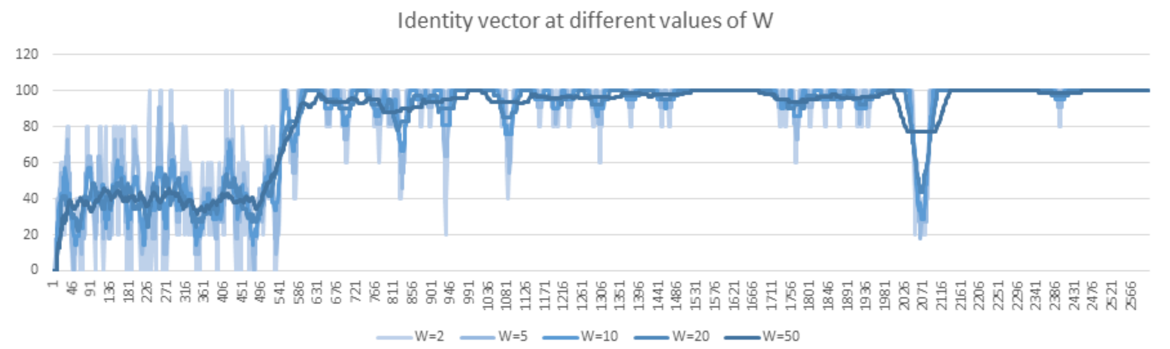

Figure 2. Identity vector using different values of W

The selection of a proper W value would be nonsense because it might change depending on the roiCSBs involved. We could also be interested on changes that happened at different frequencies. Therefore, instead of choosing a proper W value, which would mean changes at only one frequency, we build a vector containing all frequencies as follow:

$$I_V(x) = \sum_{i=0}^{N} A_i I_i(x)$$

Where

$$\sum_{i=0}^{N} A_i \ = 1$$

And

$$I_N(x) = \frac{1}{2N+1} \sum_{i=x-N}^{x+N} V_m(i)$$

In this model, N defines the maximum window to compute the percentage of identity and it also defines the start and end positions where the vector's values can be used. From 0 to 2N+1 and from 2N+1-ROI_length to ROI_length the Iv is uncompleted. Therefore, N cannot be as long as we want. It should be at least lesser than OFFSET. In practice we have observe that a value of 50 is enough to get good results.

Figure 3 shows the Iv vector.

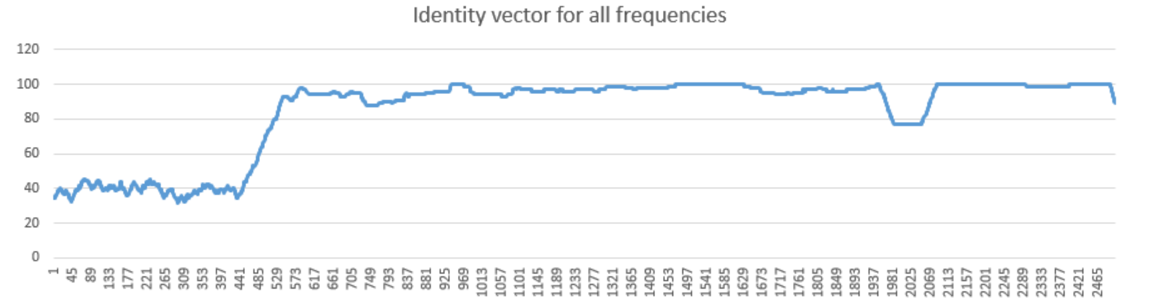

Figure 3. Identity vector using all frequencies

Calculating consensus identity vector

In the case that a group of IRs are detected, we use this information of the consensus sequence to improve accuracy of the refinement method.

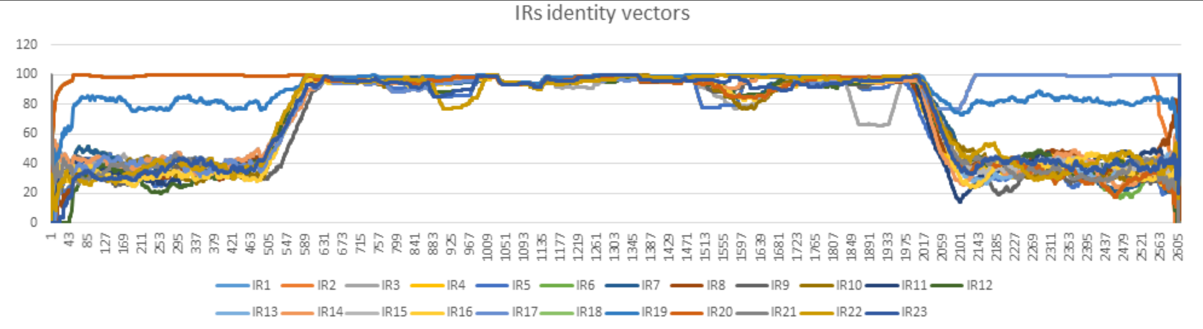

Figure 4. Identity vector for a set of Interspersed Repeats (IRs)

After IRs have been aligned and the binary vector (Vm) computed, a Sum Match Vector (SMV)is computed by adding all Vm vectors. This vector has length of ROI_length, so only positions which are not representing a GAP are taking into account -as we did in the previous section. Then, we calculate the percentage of IRs that cover one certain position in the Sum Match Vector. To calculate the Consensus Identity Vector (Icv), only positions that cover more than a threshold are setting to 1. Those below this threshold are setting to 0. This new vector is named Consensus Binary Vector. After this process, we calculate Consensus identity Vector by processing Consensus Binary Vector as we already described in the previous section.

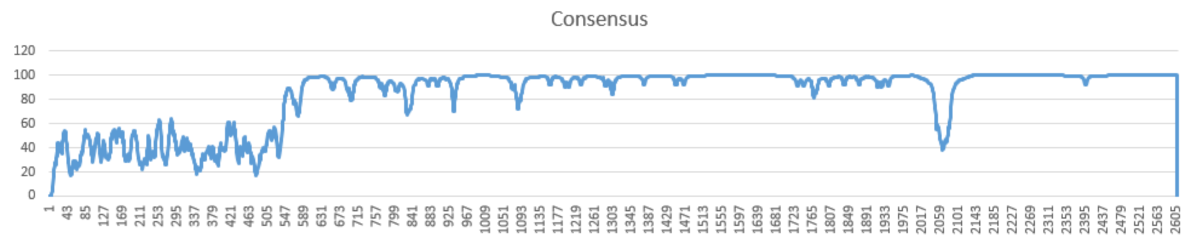

Figure 5. Consensus identity vector

Vector Difference

In order to detect transitions which delimitate the breakpoint, we compute the absolute difference between CSB's identity vector. When roiCSBs were extracted from CSBs according to the ROI, a parameter called OFFSET was introduced to make sure that homology regions would be present in roiCSBs. As a result, identity vector for the roiCSB-A have a high value at the beginning and low value at the end. On the contrary, the identity vector for the roiCSB-B have a low value at the beginning and high value at the end. This is the reason why the vector difference will start and end with high values and transitions will be in between.

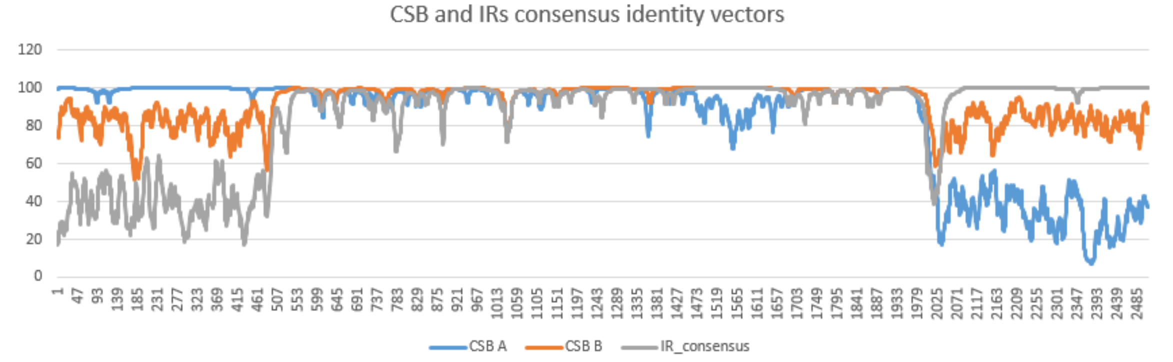

Figure 6. Identity vector for roiCSB-A , roiCSB-B and roiIRs (interspersed repeats in between)

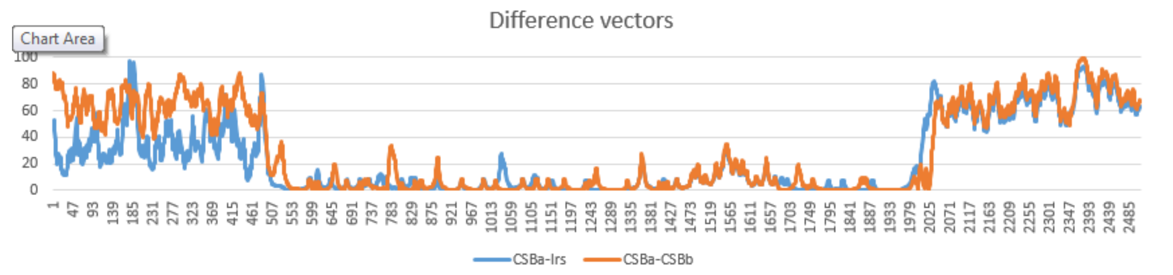

Figure 7. Vector difference

Finite State Machine

A Finite State Machine is a mathematical model of computation that has a finite number of states.

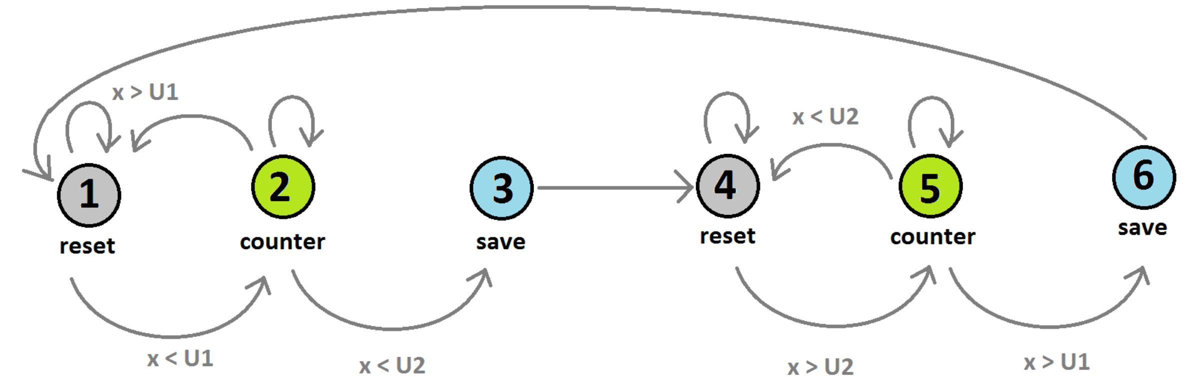

Finite State Machine to detect transitions.
This FSM has six states. First three states (1-3) are to calculate the breakpoint (BP) start coordinate, and the last three states (4-6) to calculate the BP end coordinate. To change from one state to another depends on value x (vector difference) and thresholds U1 and U2.
This FSM allows to obtain a BP as a point or region depending on the vector difference features.

FSM thresholds selection

The FSM aims to detect transitions in the vector difference. To detect these transitions, the FSM uses two thresholds, U1 and U2. To understand these two parameters, it is necessary to understand how we calculate the difference vector. In this explanation, it will be also explained the necessity of the “offset” parameter. To illustrate this explanation, we will use an example:

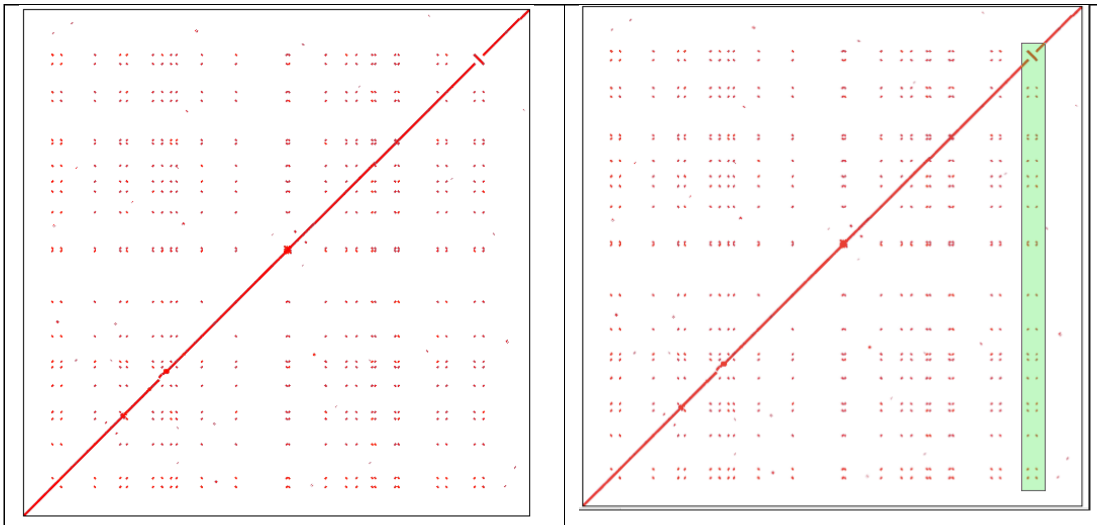

Figure 1a: Comparison
SeqX : NC_014448.1.fasta Mycoplasma hyorhinis HUB-1

SeqY : NC_019552.1.fasta Mycoplasma hyorhinis SK76

Figure 1b: Selection of CSBs and repeats involved in the rearrangement. In this special case an inversion

Once we have detected a rearrangement, we take the two CSBs involved and all the repetitions between them (see Figure 1b).

These CSBs and repeats are extended to fit the ROI region, which has been extended “offset” positions. This offset ensures that Block A and repeats will overlap at least “offset” positions in which Block A has high values of identity. The same situation happens for block B and repetitions. (see Figure 2)

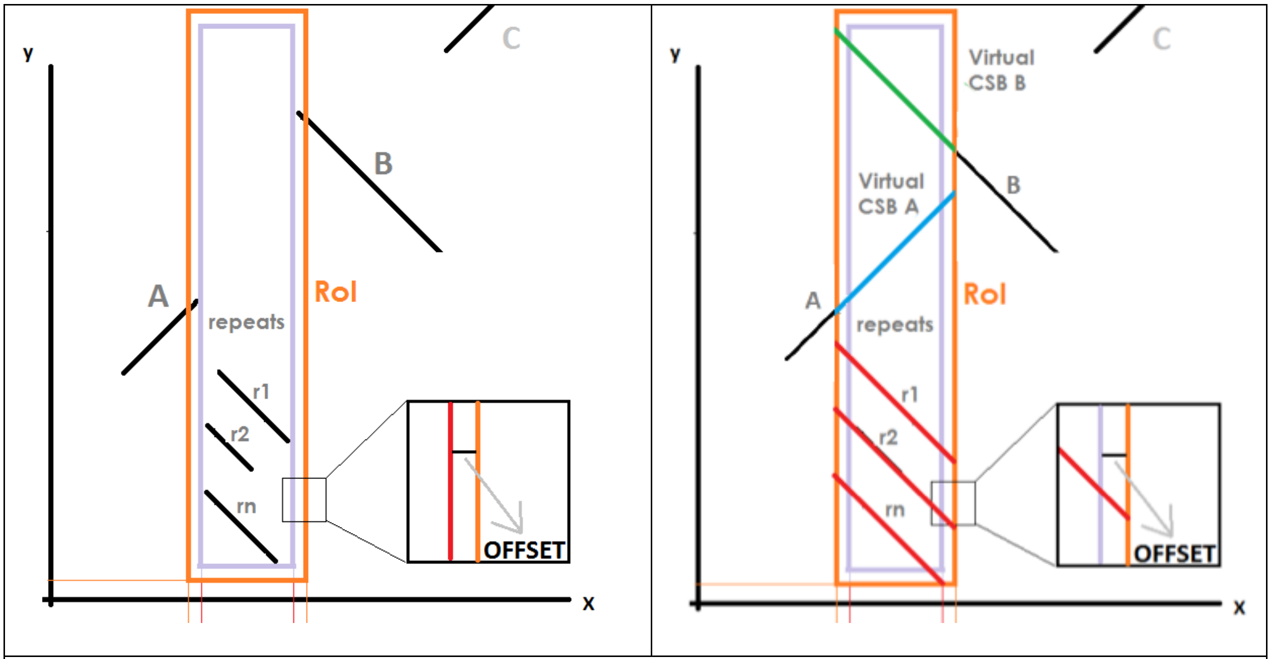

Figure 2: a) Diagram of zoomed area before extending blocks and repeats. In grey, the initial ROI. In orange, the ROI with an offset. B) Diagram after extending blocks to fit with the ROI.

After this extension of blocks, we align the virtual blocks. We only take the sequences within the ROI. Since we extended the ROI "offset" positions, we will expect high values of identity at the beginning of the Virtual CSB A (Figure 2b, blue fragment), and low values of identity at the beginning of the virtual repetitions (Figure 2b, red fragments). This process is explained formally in the method section, subsection named "Calculating the region of interest"

In the case of repetitions, we calculate a consensus first, and then we calculate the identity vector.

The way in which we calculate the identity vector is explained at "Calculating identity vectors", in method section. In summary, the identity vector is calculated as the contribution of many identity vectors for different frequencies. Using small windows (W) we get a identity vector with high frequencies, and large windows produce a identity vector with low frequencies. In the Figure 3a are represented identity vectors for virtual CSB A, virtual repetitions and virtual CSB B, using different values of W.

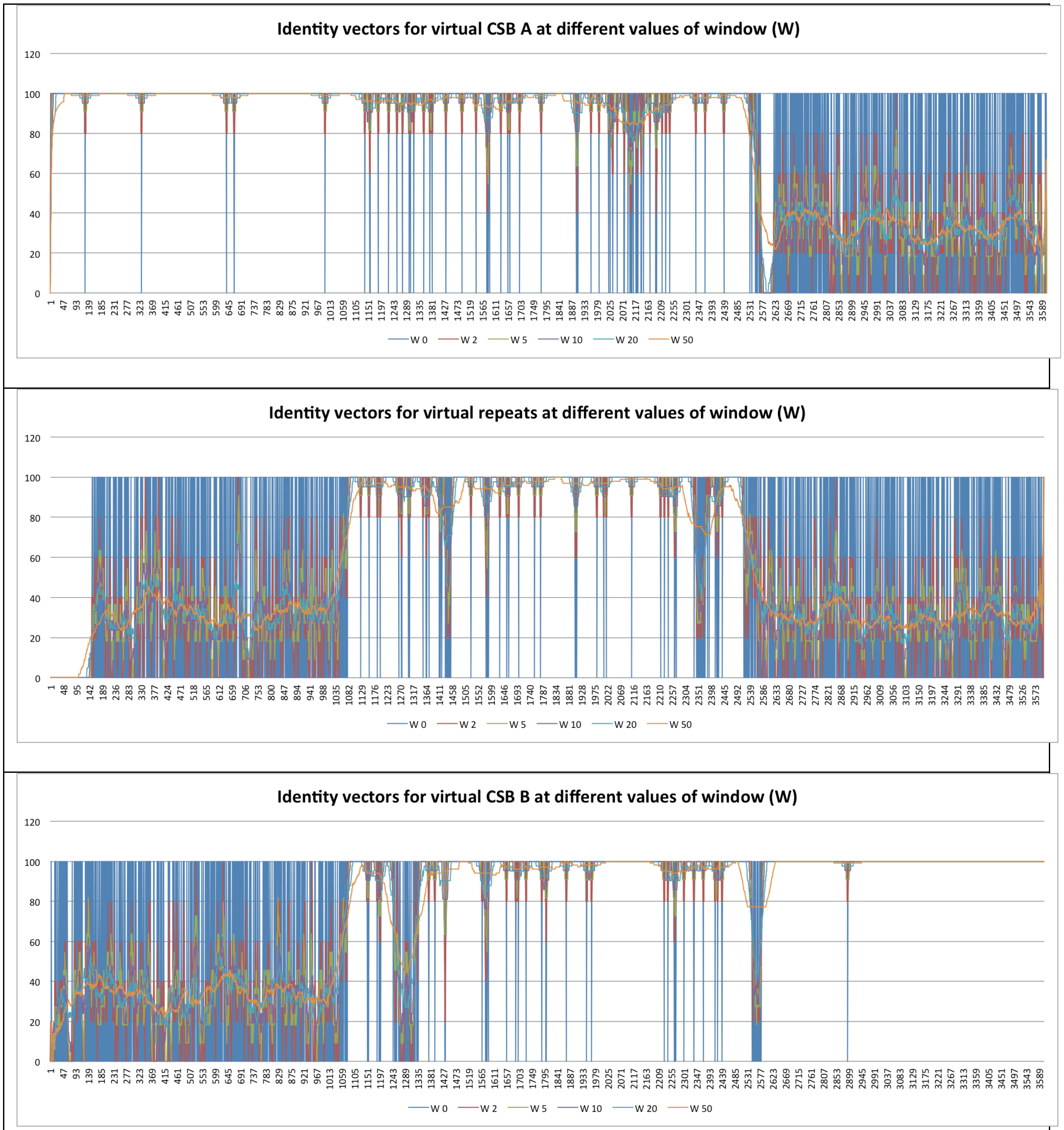

Figure 3: Identity vector using different values of window. Blue for 1, representing high frequencies, Orange for 50, representing low frequencies. On top, identity vector for virtual CSB A, in the middle for virtual repetition, on the bottom for virtual CSB B.

The final identity vector will be a contribution of different frequencies. In Figure 4 we represent the 3 identity vectors.

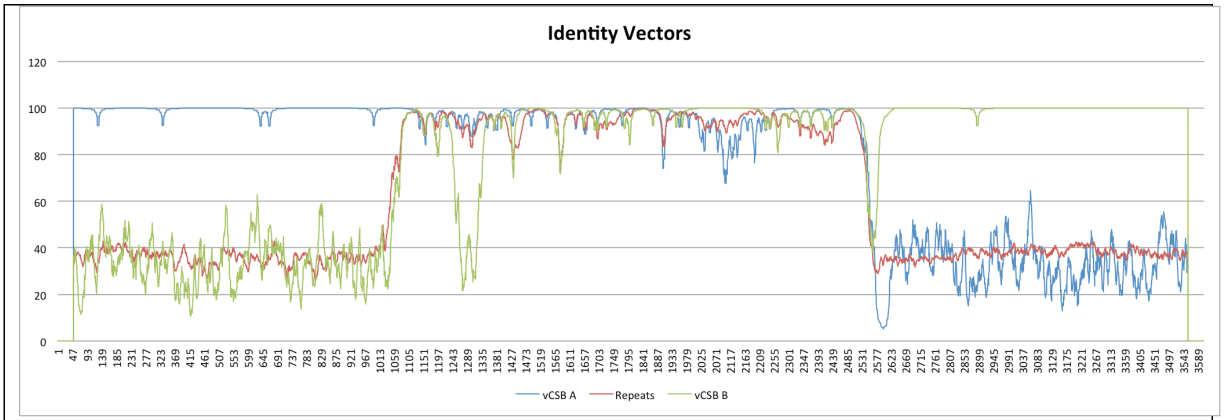

Figure 4. Identity vectors

The next step is to calculate the difference vector. This vector will emphasize the changes among identity vectors. The reason we use an offset is just to ensure that at the beginning of the virtual CSB A, high values will be present, and therefore, the vector difference will have at the beginning high values as well, where we will find the maximum, which will be used to normalize the vector. Figure 5a shows the difference vector, and Figure 5b, the normalized difference vector.

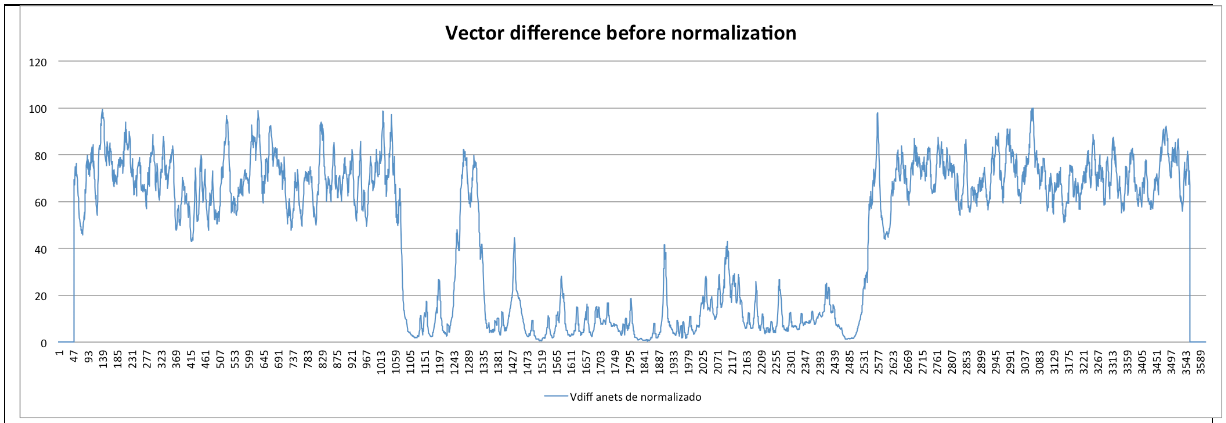

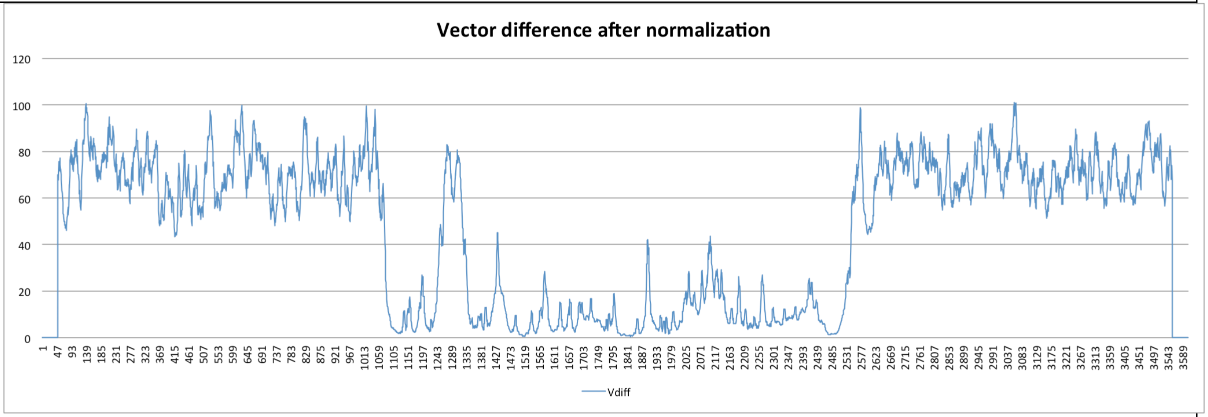

Figure 5: Vector difference before (top) and after (bottom) normalization

In this special case, the normalization process does not have a visual effect, but this process ensures that we will always have values from 0 to 100.

At this point, we apply the Finite State Machine (FSM) to detect the transitions, in order to refine the borders. The FSM needs two thresholds. One for high values and another for low values. In order to avoid the “noise” of the signal, we set empirically the thresholds at 80 and 20. Transitions detected by the FSM are showed in Figure 6.

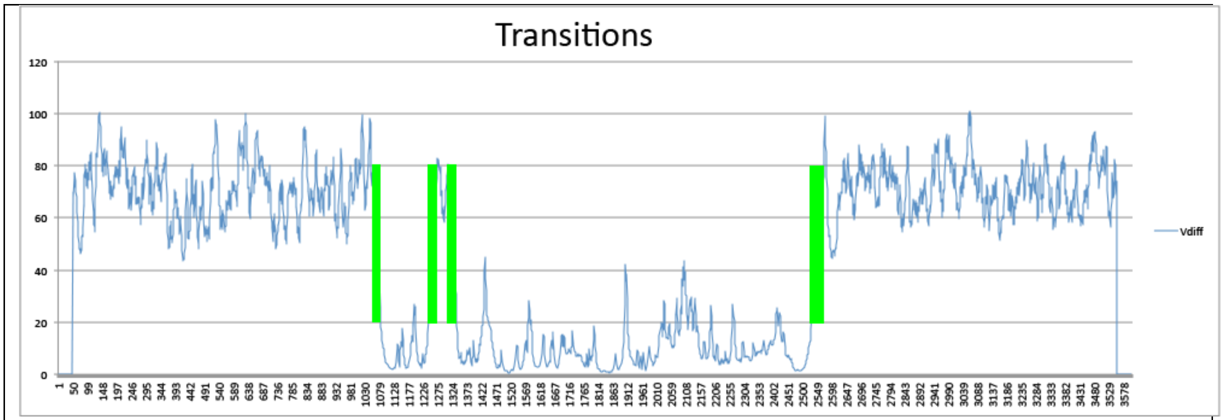

The dynamic selection of the thresholds would be itself a big problem to solve. We realized that fixed parameters actually work and a dynamic selection of the parameters might increase the accuracy. However, this dynamic selection would depend on the blocks under study and especially on the conclusion after a big study involving not only mycoplasmas but also all bacteria or even more complex organisms.

Step by Step example

Parametros (16):

-----

fasta x : ../fasta/NC_014448.1.fasta

fasta x-rev: ../fastaRever/NC_014448.1-revercomp.fasta

fasta y: ../fasta/NC_019552.1.fasta

fasta y-rev: ../fastaRever/NC_019552.1-revercomp.fasta

matFile: /home/arjona/SVN/LNCCInstallation/HSPandCSB/bin/matrix.mat

iGAP: 100

eGAP: 1

OFFSET: 2000

Refining X sequence...

ROI start: 745965 end: 751573

Num de IRs: 32

Refining Points:

2036 - 3599

Breakpoints:

2036 2047

3548 3599

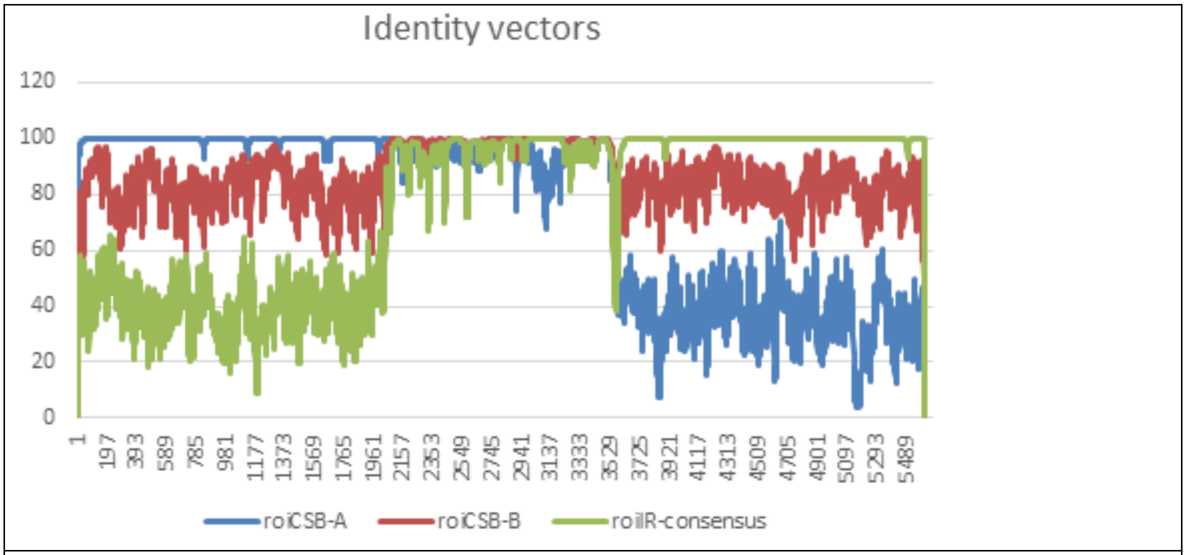

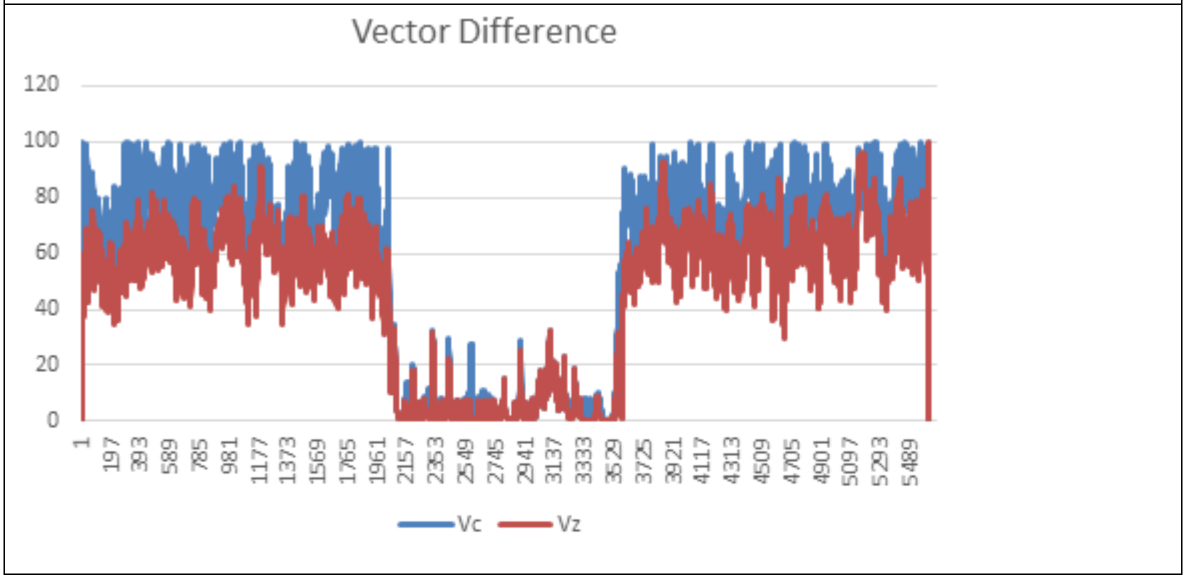

Refining Y

Number of detected points: Vc: 8

Lnegth vectors: 5633

1740	2086	3018	3033	3048	3072	3593	3876
2038	2086	3018	3029	3053	3072	3591	3603

Refined points:

2038 - 3603

Breakpoints:

2038	2086

3591 3603

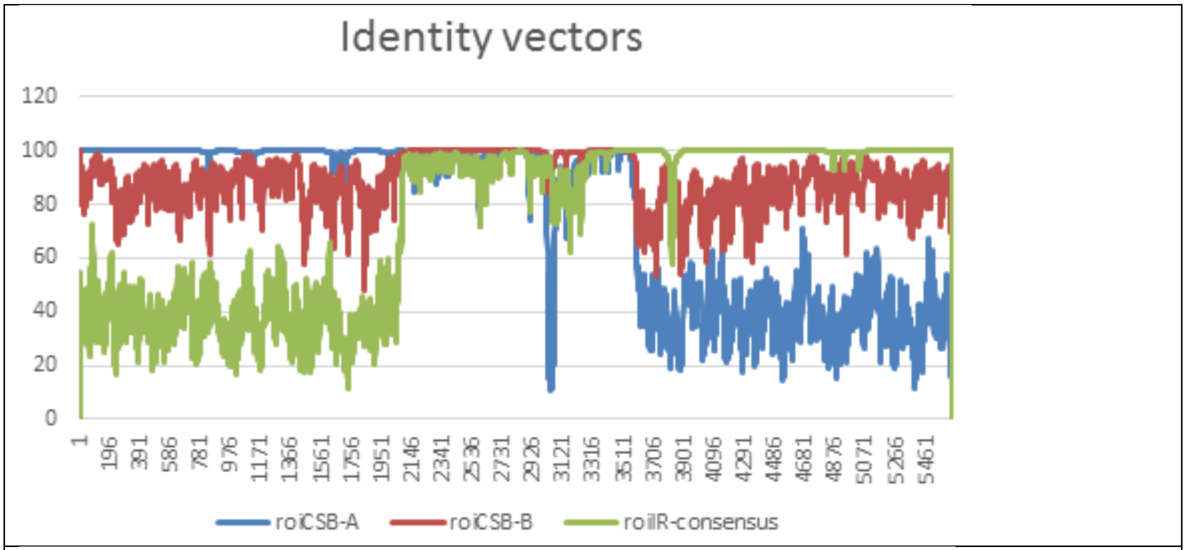
Identity vectors
120
100
80
60
40
20
0
1
196
391
586
781
976
1171
1366
1561
1756
1951
2146
2341
2536
2731
2926
3121
3316
3511
3706
3901
4096
4291
4486
4681
4876
5071
5266
5461
roiCSB-A
roiCSB-B
roiIR-consensus

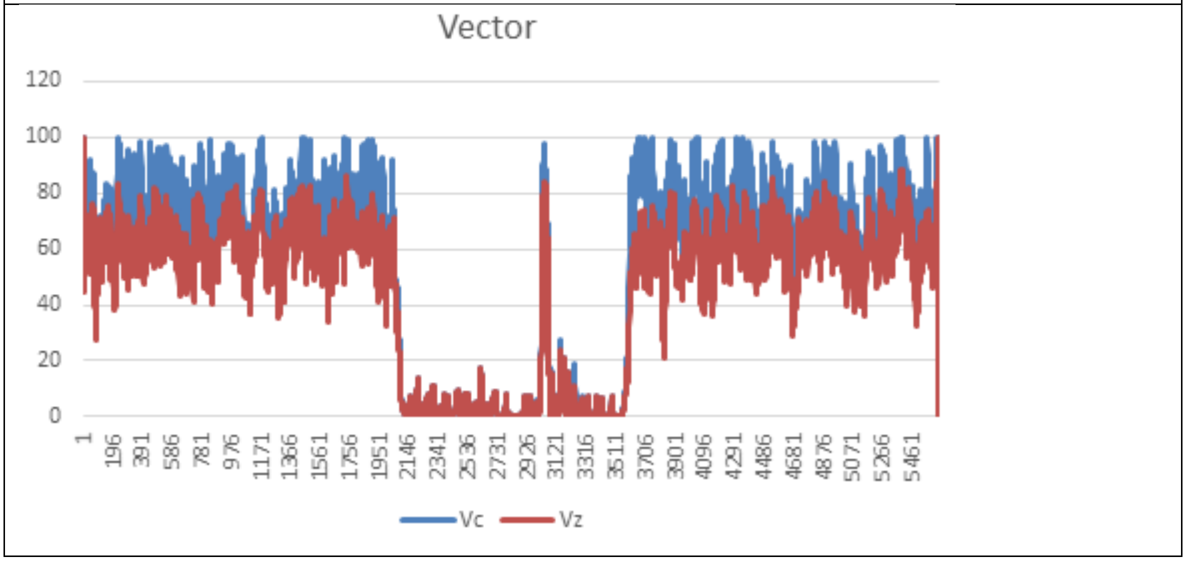
Vector
120
100
80
60
40
20
0
1
196
391
586
781
976
1171
1366
1561
1756
1951
2146
2341
2536
2731
2926
3121
3316
3511
3706
3901
4096
4291
4486
4681
4876
5071
5266
5461
Vc
Vz
